# The use of a whole inactivated PRRS virus vaccine administered in sows and impact on maternally derived immunity and timing of PRRS virus infection in piglets

**DOI:** 10.1002/vro2.34

**Published:** 2022-04-05

**Authors:** Gerard Eduard Martín‐Valls, Preben Mortensen, Hepzibar Clilvert, Yanli Li, Martí Cortey, Melanie Sno, Timea Barna, Marisa Terré, Nicolas Guerra, Enric Mateu

**Affiliations:** ^1^ Departament de Sanitat i Anatomia Animals Facultat de Veterinària Travessera dels Turons s/n Universitat Autònoma de Barcelona Barcelona Spain; ^2^ Ceva Santé Animale Libourne France; ^3^ Ceva Animal Health Ceva Phylaxia Veterinary Biologicals Co. Ltd., Budapest Hungary; ^4^ Cooperativa d'Artesa de Segre Artesa de Segre Lleida Spain; ^5^ C ReSA‐IRTA‐UAB Campus UAB Cerdanyola del Vallès Barcelona Spain

## Abstract

**Background:**

Porcine reproductive and respiratory syndrome virus (PRRSV) vaccination is usually based on administering periodically PRRS modified live virus (MLV) in sows throughout their life. Using this schedule, transfer of maternally derived antibodies to the offspring is limited. The aim of the present study was to test the concept of priming with an MLV and boosting with a commercial inactivated virus vaccine in sows to reduce PRRSV incidence and improve productivity.

**Methods:**

On two farms, all the sows were vaccinated with a MLV vaccine at week 8 of gestation. Then two groups were designated, one group was re‐vaccinated in the third week prior to farrowing and using a commercial inactivated vaccine (the PG group). The second group was the control group (C). Assays for PRRSV infection and productive parameters were evaluated.

**Results:**

For both farms, the incidence of PRRSV was lower at 6 weeks of age in PG than in C (*p* < 0.05). At weaning the proportion of PRRSV seropositive piglets was higher for PG as well (*p* < 0.05). The litters from C sows from both farms showed a higher pre‐weaning mortality (odds ratio, C vs. PG = 1.18 ± 0.09; *p* < 0.05).

**Conclusions:**

Administration of the vaccine in sows before farrowing was safe and associated with reduced incidence of PRRSV in piglets up to 6 weeks of age.

## INTRODUCTION

Porcine reproductive and respiratory syndrome (PRRS) is characterised by abortion, respiratory disease, increased piglet mortality^1^, ^2^, ^3^, ^4^ and secondary infections.^5^, ^6^, ^7^ In a previous study,^8^ PRRS cost about US$ 664 million in the USA; weaners and growers accounted for 55% of the cost. In Europe, the average cost during an outbreak has been estimated at €126 per sow.^9^

Transmission of PRRSV may occur by several routes including direct contact with fomites.^10^ Current vaccines do not produce sterilising immunity and transmission to or from vaccinated pigs is possible. Controlling PRRS without vaccines is difficult. On most farms, the goal is to stop virus circulation among breeding pigs (stabilisation).^11^ Such control programmes may start with administration of a modified live vaccine (MLV) for a primary immunisation.^12^ Once stabilisation is achieved, eliminating PRRSV in the piglet nursery is feasible by depopulation. Vaccination of piglets is an option for reducing PRRSV transmission.^13^, ^14^ However, vaccination of piglets interferes with monitoring and requires additional measures to avoid recombination between field and vaccine strains.

Whole PRRSV inactivated vaccines (IV) can be useful to boost previous immunity. This approach is appealing when aimed at increasing the levels of maternally derived antibodies (MDA). Passively transferred neutralising antibodies (NA) may protect against the development of viraemia.^15^ Strategies resulting in increased MDA transfer may contribute to delaying the circulation of PRRSV and minimise the impact of infection and disease. The present study explores this concept by using an IV before farrowing, after priming sows in the eighth week of gestation with a MLV. The efficacy of this vaccination scheme was assessed by comparing incidences, antibody levels and production parameters of piglets born from sows vaccinated with the combination of an IV with an MLV compared with piglets born from sows receiving only the MLV.

## MATERIALS AND METHODS

### Selection of the farms and design of the study

Two farrow‐to‐wean farms where PRRSV circulated in the farrowing units were included in the study. The circulating PRRSV strains were sequenced (whole genome; accession MZ318698 and MZ318699). Farm 1 (300 sows) was run on a 3‐week batch basis while Farm 2 (1700 sows) operated on a weekly basis. Both farms obtained PRRSV‐free gilts of about 6 months of age that were quarantined until the first service. Gilts were immunised with two doses (4 weeks apart) of a PRRS MLV‐Porcilis PRRSV (MSD) for Farm 1 and Pyrsvac‐183 (Syva) for Farm 2 before the first insemination. These farms had two physically separated farrowing units (≥14 sows) and this enabled at least two separated flows of weaners (all‐in/all‐out, no shared airspace). Farm 1 weaned pigs at 28 days and Farm 2 at 21 days. On both farms, sows were blanket vaccinated with the MLV vaccine three times a year.

Before the beginning of the trial, all sows were vaccinated once with the regularly used MLV in the farms to provide a baseline for their immune status.

**FIGURE 1:**
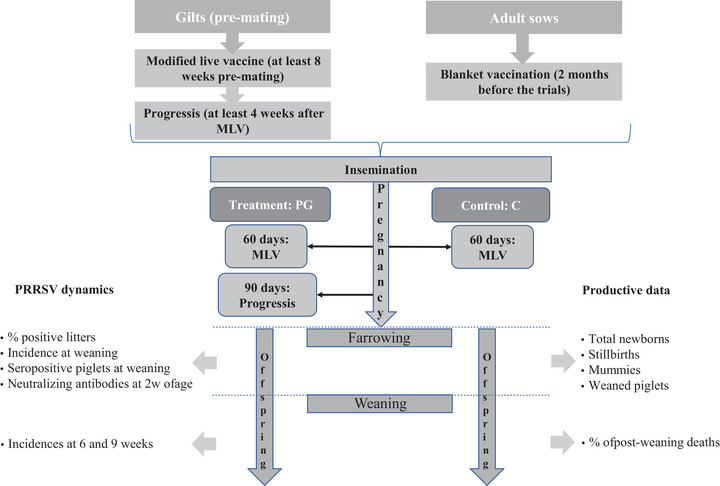
Graphical description of the study design

The design of the study (Figure 1) was to produce a treatment (PG) and a control group (C). On both farms and for all batches, piglets in half of the farrowing rooms were weaned in a nursery unit and those from the other half were weaned in a separated unit. One half of the sows were allocated to the treatment group (PG) and the other half to the control group (C). All sows were administered the MLV at the eighth week of pregnancy and were either re‐vaccinated with the IV (PROGRESSIS, group PG) 4 weeks before the expected farrowing date (around 90th day of gestation) or did not receive any other booster (group C). Parity was matched as much as possible between PG and C sows. Six batches of piglets were followed on Farm 1 (representing 77 PG sows and 78 C sows) and three batches on Farm 2 (77 PG and 71 C sows). On Farm 1, only the gilts in the sixth batch in the PG group received a dose of IV before the first insemination; that is, 4 weeks after the last MLV dose in quarantine and, at least, 4 weeks before insemination. On Farm 2, all gilts in the PG group received the IV before the first insemination, similarly to the sixth batch of Farm 1.

### Sampling

Umbilical cords (UC) were collected at birth from 6 piglets/litter (7–8 for the sixth batch of Farm 1) that were ear‐tagged. To avoid bias, cross‐fostering was limited to the minimum possible to assure survival of piglets (only during the first 24 h of life). The animals that were cross‐fostered were identified by an ear‐tag and were excluded. Animal movements between farrowing crates were recorded. On Farm 1, 155 litters were examined (77 PG and 78 C sows, with 785 newborn piglets, 473 PG and 480 C followed for the whole study period). On Farm 2, 148 litters were examined (77 PG and 71 C sows, with 826 newborn piglets, 448 PG and 418 C piglets followed).

At weaning, ≥3 piglets/sow (among those where UC had been collected) were blood sampled in order to compare ≥45 animals/group/batch (to evaluate a 50% reduction in prevalence, considering a 50% basal prevalence per batch at weaning, with 95% confidence and 80% power). The hypothesis was that the proportion of PRRSV‐infected animals in the PG group would be half of the proportion in group C (95% confidence). To evaluate the PRRSV incidence in nurseries at least 30 pigs/group among those sampled at weaning were sampled again at 6 and 9 weeks of age (WOA) (50% reduction of positive animals per batch, 95% confidence, 80% power, considering an initial prevalence of 70% at 6 WOA). Table 1 summarises the number of animals followed up per age, group, batch and farm.

**TABLE 1:** Samples collected per farm, batch and age/type

			Number of piglets			
Farm number	Batch number		Umbilical cord	4 weeks	6 weeks	9–10 weeks
Farm 1	# 1	PG	96/16	51	43	43
		C	96/16	53	31	31
	# 2	PG	78/13	50	30	30
		C	84/14	50	29	29
	# 3	PG	84/14	47	46	46
		C	84/14	49	49	49
	# 4	PG	84/14	65	32	31
		C	84/14	50	32	32
	# 5	PG	84/14	48	46	42
		C	84/14	48	44	40
	# 6	PG	47/6	45	44	44
		C	48/6	47	46	46
	Total	PG	473/77	308	241	236
		C	480/78	295	231	227
Farm 2	# 1	PG	126/21	83	83	82
		C	126/21	85	85	83
	# 2	PG	126/21	85	85	87
		C	126/21	85	85	75
	# 3	PG	196/35	100	99	87
		C	166/29	87	85	75
	Total	PG	448/77	268	267	254
		C	418/78	257	255	243

*Note*: PG offspring of sows receiving the whole virus inactivated vaccine. C offspring of control sows. The column ‘umbilical cord’ shows the number of samples collected/number litters examined.

### Analysis of the samples

Umbilical cords were homogenised and pooled in pairs. For positive pools, individual samples were examined afterwards. Sera were analysed individually.

RNA was extracted with the kit MagMax Core nucleic acid purification kit (Life Technologies). RT‐qPCR for the detection of PRRSV was performed using the LSI VetMax PRRSVEU/NA v2.0 kit (Life Technologies). According to manufacturer instructions, Ct values <40 were considered positive.

Antibodies were analysed with a commercial ELISA (Idexx PRRSV X3). Animals were analysed at weaning and 9 WOA. Animals that tested negative by RT‐qPCR throughout the study and were positive by ELISA at 9 WOA were considered to have been infected.

A vertical transmission event was considered to have happened when at least one UC per litter tested positive by RT‐qPCR. At weaning, 6 and 9 WOA, the proportion of positive animals was calculated based on the RT‐qPCR results. Incidence was calculated considering the number of susceptible animals (S) and new cases (NC) for each sampling period. Cumulative incidence was calculated by aggregating the data between sampling periods (weaning to 6 weeks, 6–9 weeks). An animal found to be infected in two consecutive samplings was considered as a NC only in the first positive sampling time. Animals seroconverting at 9 WOA were added as NC for this sampling time‐point. Only the animals that could be followed throughout the study were considered for these calculations. The results obtained at birth were compared with the appearance of RT‐PCR‐positive pigs at weaning to evaluate if transmission chains had occurred.

Sera were selected and evaluated by the viral neutralisation test (VNT) using the MLV of each farm as antigen.^16^ On Farm 1, at least 23 sera/group were collected before weaning in batches 1–4. In batch #6, a further 23 extra piglets/group were randomly selected. For Farm 2, 49 and 52 sera from PG and C groups were randomly selected from batches 1 and 2.

### Reproductive data

The following data were collected. (1) Total piglets born per litter, the number of born alive piglets per litter, stillbirths per litter, mummified per litter and weaned piglets per litter and (2) mortality in the nurseries. These data were obtained for all sows in each batch (*n* = 89 for PG and *n* = 72 for C sows and their respective progenies on Farm 1; *n* = 131 for PG and *n* = 135 for C sows on Farm 2) and not only for the ones included in the PRRSV follow‐up.

### Statistical analysis

To assess the effect of vaccination on PRRSV incidence (weaning to 6 weeks and 6–9 weeks) a generalised linear mixed‐effects model (GLMM) in Rstudio Cloud (glmer function; emmeans, Matrix and lme4 libraries) was used. Treatment (IV vaccination vs. non‐vaccination) and farm were considered fixed effects; the batch was considered as a random effect nested on the farm. Cumulative incidences from 4 to 6 and from 6 to 9 WOA and the proportion of seropositive animals at weaning were aggregated per batch and compared between vaccination groups per farm using a chi‐square test with Yates correction. Relative risk was calculated using the Koopman's likelihood‐based approximation.

Neutralisation titres (log_2_ converted for normalisation of the data) were compared between PG and C groups by the ANOVA and Kruskal–Wallis tests (non‐normally distributed variables) on StatsDirect 3.2.10.

For reproductive data, a GLMM was used considering the data as binomial. In this model, pre‐weaning mortality in a litter could be influenced (fixed effects) by the treatment, the number of live piglets that were born and the parity of the sow. The interaction between the treatment and the number of piglets born alive was included since vaccination could prevent vertical transmission. Data were transformed logarithmically. Batch was used as a random effect. Since GLMM does not calculate the *p*‐values, these were obtained using the Wald chi‐square test (car library) and likelihood ratio tests (drop1 function).

## RESULTS

### Dynamics of PRRSV circulation as assessed by RT‐PCR

Before the trial, PRRSV was detected at weaning by RT‐qPCR, confirming that the farm was infected. In the following text, the results are firstly summarised per farm and batch and then are aggregated in the GLMM.

### Comparison of PRRSV incidences between PG and C groups

Generalised linear mixed‐effects model showed that, for both farms, from 4 to 6 WOA, the PRRSV incidence was lower in the offspring of PG sows compared to the C group (*p* = 0.014). From 6 to 9 weeks, after fading of MDA, the incidence was higher in the offspring of PG than in C (*p* = 0.044) (see Table 2).

**TABLE 2:** Output of the generalised linear mixed models used in the present work for the comparison of productive data and incidences

	Evaluated variables	Estimate	Standard error	*z*‐Value	Pr(>|*z*|)	*F*‐value	*p*‐Value (chi)
Mortality	All batches included						
	*Intercept*	−5.51	0.670	−8.225	<2e‐16		
	*GROUPV*	−0.73	0.940	−0.773	0.440		
	*log(ALIVE)*	1.45890	0.250	5.834	5.41e‐09		
	*log(PARITY)*	0.00191	0.047	0.040	0.968		
	*GROUPV:log(ALIVE)*	0.28830	0.353	0.816	0.414		
	Batch 5 from Farm 1 excluded						
	*Intercept*	−6.116	0.739	−8.268	<2e‐16		
	*GROUPV*	−3.003	1.070	−2.805	−0.005	0.136	0.005
	*log(ALIVE)*	1.706	0.274	6.205	5.45e‐10	121.72	5.453e‐10
	*log(PARITY)*	−0.024	0.051	−0.483	0.628	0.083	0.628
	*GROUPV:log(ALIVE)*	1.097	0.400	2.741	0.006	7.504	0.006
PRRSV incidence	Incidence 4–6 weeks (both farms)						
	*Intercept*	−2.09089	1.09	−2.662	0.007		
	*GROUPV*	−1.6307	0.66	−2.446	0.014	5.774	0.0144
	Incidence 6–9 weeks (both farms)						
	*Intercept*	−1.086	0.691	−1.572	0.115		
	*GROUPV*	0.314	0.156	2.017	0.043	4.076	0.0436

*Note*: GROUPV represents PG group, log(ALIVE) represents born alive, log(PARITY) represents the parity of sows, and GROUPV:log(ALIVE) represents the interaction between both variables. For productive data, only when batch #5 from Farm 1 was excluded it was possible to observe an effect of the vaccination group, which resulted in a reduction of the mortality for PG group (GROUPV). In the case of the incidences, the estimates indicate that that the PRRSV incidence was lower for PG group (negative value for GROUPV) before 6 weeks of age, and vice versa from 6 to 9 weeks.

### Detailed PRRSV dynamics for Farm 1

Regarding the UC results, 12.7% (8/63; confidence interval 95% (CI_95%_): 5.6%–23.5%) of the PG litters had positive results with an average Ct of 34.5 ± 3.4. In C, 10.9% of the litters had positive RT‐qPCR results (7/64, CI_95%_: 4.5%–21.2%, non‐significant) with an average Ct of 36.5 ± 3.1. Only 2/15 litters tested positive by RT‐qPCR at weaning (one PG and one C).

**FIGURE 2:**
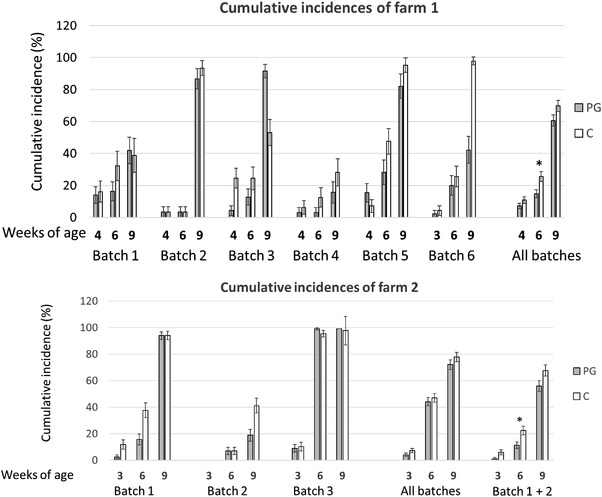
Cumulative incidence at weaning (3–4 weeks), 6 and 9 weeks of age observed per batch for Farms 1 and 2. Grey bars correspond to PG group and empty bars to C group. *Significative differences at 6 weeks of age, where PG group showed lower incidence from 4 to 6 weeks of age

For Farm 1, 467 pigs were followed from birth until 9 WOA (236 PG and 231 C). Cumulative incidence until 6 WOA was 25.5% in C group and 14.8% of piglets in group PG tested positive (*p* < 0.05), resulting in a relative risk of 1.57 (CI_95%_: 1.07–2.29). Piglets in group C were 1.57 times more likely to be viraemic than piglets in the PG group. A trend for lower Ct values in infected piglets of group PG was observed (29.7 ± 5.8 for C vs. 32.6 ± 4.8 for PG, *p* = 0.0503). The maximum incidence observed was at 9 WOA in both groups. Figure 2 shows the evolution of the cumulative incidence of each group.

### Detailed PRRSV dynamics for Farm 2

For Farm 2, circulation of PRRSV was also detected at weaning before the beginning of the trial. When examining UC, 14.3% of the PG litters (11/77; CI_95%_: 7.3%−24.1%) and 8.4% (6/71; CI_95%_: 3.2%−17.5%) of the C litters contained PRRSV‐positive UC (non‐significant). The PG and C UC samples yielded similar Ct values (37.9 vs. 37.5). On this farm, 1/11 litters in the PG group had RT‐qPCR‐positive piglets at weaning.

On this farm, the cumulative incidences at 3, 6 and 9 WOA were similar in all groups (see Figure 2). These results were affected by increased circulation of the virus at 6 WOA in the third batch (>97% incidence) in both PG and C piglets. Since the behaviour of the infection in the third batch differed substantially from that of previous batches in this farm or Farm 1, this batch was considered to be an outlier. Considering only the first two batches, the cumulative incidence at 6 WOA was significantly lower in PG group (11.3%; CI_95%_: 6.9%−17.1% vs. 22.4%; CI_95%_: 16.1–28.6 of incidence; *p* < 0.05) resulting in a relative risk of 1.9 (CI_95%_: 1.2–3.3; *p* < 0.01) for C versus PG. The Ct values were similar in both groups (32.7 ± 3.2 for C vs. 34.3 ± 2.9 for PG). Figure 2 shows the incidences for Farms 1 and 2 for the aggregated batches. Supporting Information S1 shows the individual results per batch.

### ELISA and viral neutralisation test

#### Farm 1

**FIGURE 3:**
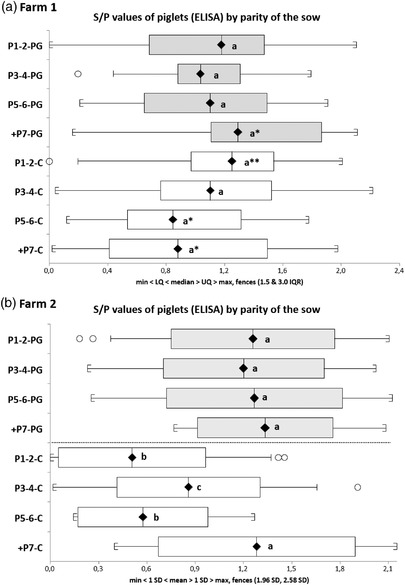
Distribution of *S*/*P* ratios by parity of the sow in Farm 1 (batches 1–4 and 6) and Farm 2 (batches 1 and 2). The whiskers and boxes depict, from left to right: minimum, lower quartile, median (diamond), upper quartile and maximum. The *Y*‐axis indicate the parity of the sows in PG and C groups. Statistically significant differences (*p* < 0.05) are shown by different letters between groups within each farm. Asterisks indicate a *p*‐value between 0.1 (*) and 0.05 (**)

On Farm 1, the proportion of seropositive (ELISA) piglets at weaning in the offspring of PG sows was 94.1% versus 86.5% in C piglets (*p* < 0.01). This corresponded to a relative risk of 2.4 (CI_95%_: 1.3–4.4; *p* < 0.01) indicating that PG animals were more likely to have anti‐PRRSV MDA at that age. When comparing the average *S*/*P* ratios for the whole population or only for the seropositive piglets, no significant differences were observed (1.08 ± 0.45 vs. 1.04 ± 0.50 in PG and C for all animals; 1.13 ± 0.42 for PG vs. 1.16 ± 0.45 for C if only seropositive animals were considered). When the *S*/*P* ratios were examined with regards to the parity of the sow, the oldest PG sows tended to have higher *S*/*P* ratios than C sows, except those of parities 3 and 4 (Figure 3).

**FIGURE 4:**
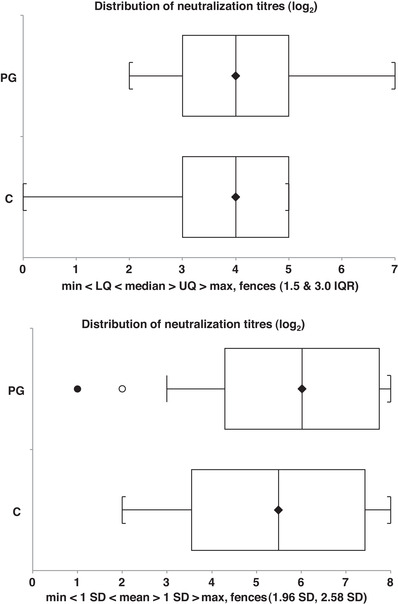
Distribution of the neutralisation titres in the pre‐weaning piglets per group in Farm 1 (upper) and Farm 2 (lower). For Farm 1, batches 1–4 are shown (no pre‐mating vaccination in primiparous sows) and for Farm 2 batches 1 and 2. Whiskers and boxes show minimum, lower quartile (25%), median, upper quartile (75%) and maximum. Titres are shown as log_2_ values

Regarding the VNT before weaning, the average log_2_ titre for PG was 3.6 ± 0.2 versus 3.5 ± 0.4 for C group (non‐significant). The dispersion of titres was different in the vaccinated and control groups (Figure 4). Titres >5log_2_ were only observed in the offspring of PG sows (Supporting Information S2).

**FIGURE 5:**
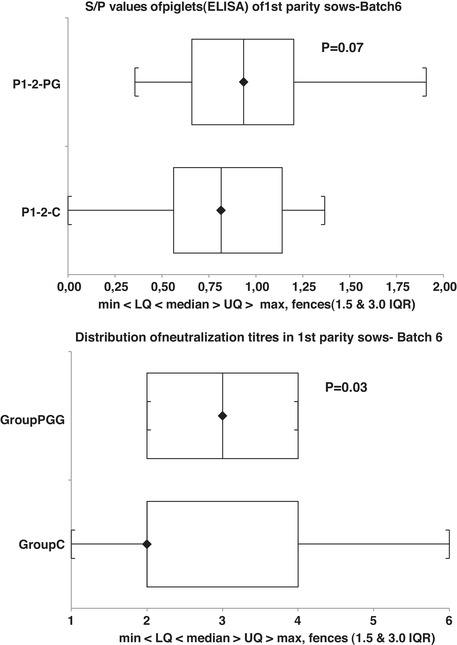
*S*/*P* ratios (a) and neutralisation titres (log_2_) (b) per group (PG and C) in the offspring of parity 1 (P1) sows of Farm 1. Whiskers and boxes show minimum, lower quartile (25%), median, upper quartile (75%) and maximum. PG piglets tended to have higher *S*/*P* ratios at weaning than C sows (*p* = 0.09)

Gilts from the sixth batch were boosted with the IV after priming and before mating. They then received a second IV dose before farrowing. Comparison of the MDA levels in the offspring showed that *S*/*P* ratios and NA titres tended to be higher in the PG group (*p* = 0.08 and 0.03, respectively) (Figure 5). The distribution of the NA titres in the PG group was more homogeneous than in the C group.

#### Farm 2

When considering only the two first batches, at weaning 158/168 (94.1%; CI_95%_: 90.5%–97.1%) animals were positive in the PG group and 129/170 (75.9%; CI_95%_: 69.5%–82.3%) were positive in the C group (*p* < 0.0001). Considering all batches, the proportion of seropositive piglets in the offspring of PG sows was higher (74.9% vs. 59.9%, for PG and C, respectively, *p* = 0.02). No differences were observed when comparing *S*/*P* ratios for positive animals considering either batches #1 and #2 or the three batches. When the distribution of *S*/*P* ratios in positive piglets only was examined, regarding the parity of the sow, differences were clear (Figure 3). Thus, in the PG group, *S*/*P* ratios were similar in all piglets regardless of the parity of their mothers. While in the C group the *S*/*P* ratios of the offspring of young sows (parities 1 and 2) were lower than the average *S*/*P* ratios of older sows (parity ≥7) or than the *S*/*P* ratios of piglets born from PG sows of any parity (*p* < 0.05).

The VNT was carried out only with seropositive animals (ELISA) in batches 1 and 2. The average log_2_ titre of MLV NA for PG animals was 6.0 ± 1.7 versus 5.5 ± 1.9 in C animals (*p* = 0.08) (Figure 4). When the titres were considered as ‘high’ (≥1:32) or ‘low’ (1:4 to 1:16) some 83.0% of the PG piglets had high titres while 63.3% of the C piglets had them (*χ* ^2 ^= 5.1; *p* < 0.05).

### Reproductive data

On Farm 1, suckling piglets in batch #5 suffered an outbreak of diarrhoea. The outbreak started in a room where PG sows were present. During the outbreak mortality reached 13% and 24% for C and PG groups, respectively. The RT‐qPCR analysis of batch #5 piglets (2 and 4 WOA) showed that PRRSV‐positive animals belonged to three litters in each group. None of the PCR‐positive piglets of batch #5 died before weaning. Since the outbreak started in the PG group and was associated with higher mortality in that group, this batch was biased by this event. As the present trial was designed as an intention‐to‐treat study and batch #5 was biased by a cause external to the treatment affecting some of the assessed outcomes, batch #5 violated the protocol and was removed from the calculations. Even so, in terms of MDA and PRRSV incidence at 6 and 9 WOA, batch #5 performed as the other five batches. Results for batch #5 are presented in Supporting Information S3.

No differences were observed regarding the number of newborn, stillborn or mummified piglets per litter. The GLMM analysis including all batches on both farms did not show a significant impact on the survival of suckling piglets. When batch #5 was removed (because of the diarrhoea outbreak) the results suggested that vaccination had a significant effect on reducing mortality (see Table 2). The effect was estimated with an odds ratio of 1.18 ± 0.09 (*p* = 0.0405) (C vs. PG). Mortality was also related to the number of born alive piglets and its interaction with the treatment. This interaction was an essential variable of the GLMM as calculated with the likelihood ratio test (*p* = 0.00661). Parity had no significant effect. Supporting Information S3 shows the reproductive data. No significant differences were observed regarding mortality in nurseries (Supporting Information S4).

## DISCUSSION

The impact of PRRS virus‐associated disease in piglets will depend on several factors including the flow of viraemic piglets from farrowing units to nurseries and this can result in higher costs. Therefore, reducing the transmission of PRRSV in the farrowing units and nurseries is crucial for controlling the disease. Most often this is intended by vaccination programmes for sows, although other systems such as herd closure may be applied.^17^, ^18^ Nevertheless, on some farms, intensive vaccination programmes for sows are not sufficient. In such instances additional actions such as vaccination of piglets may potentially reduce transmission of PRRSV1.^13^, ^14^ Although MDA may partially block vaccination.^19^, ^20^

Another strategy would be to increase the NA titres against PRRSV by passive transfer. From previous studies,^15^ 1:8 to 1:16 NA titres are reported to protect piglets in the homologous model challenge. Therefore, theoretically increasing anti‐PRRSV MDA might help to decrease the incidence of PRRSV at earlier ages. It could be contented that a recall pre‐farrowing vaccination would be helpful for that purpose. The IV produces immunity when administered repeatedly.^12^ The safety of such vaccines makes them candidates for a MDA boosting strategy.

In our study on both farms the offspring of PG sows had a higher proportion of ELISA‐positive animals, suggesting that anti‐PRRSV humoral immunity was boosted. The strategy resulted in the homogenising of the antibody values in the progeny of treated sows. On Farm 1, the offspring of primiparous PG sows tended to have a NA titre that was 1log_2_ higher than the control gilts. On Farm 2, these differences could be observed for all parities and the number of piglets showing titres over 5log_2_ was significantly higher in the PG group.

The effect of vaccination on the antibody response seemed to be higher on Farm 2 than Farm 1. This may have several explanations. The MLVs used on Farms 1 and 2 and the circulating strains were different. This could lead to different interactions with the IV, resulting in different boosting of the humoral response. It is known that neutralising cross‐reactivity is extremely diverse.^21^ It would have been interesting to evaluate the impact of the vaccination strategy on the potential neutralisation of the field strains; unfortunately, it had not been possible to adapt those strains to MARC‐145 cells, therefore the MLV strains were used. Also the management on Farms 1 and 2 was different, which could lead to different policies in the farrowing units with different colostrum intake and consequently different antibody titres.

It is worth noting that in most, but not all, of the examined batches, the incidence of PRRSV at 4 or 6 WOA was lower in the PG pigs. This would agree with the previous observation of a higher proportion of seropositive animals at weaning and suggests a beneficial effect of the pre‐farrowing vaccination with an IV in delaying the spread of the virus at earlier ages. After the fade out of the MDA, animals were infected at a similar rate. Thus, when MDA disappeared the incidence increased in the PG group. This observation is interesting because it is thought that the older the animal, the lower the impact of PRRSV infection.^22^ Even so, no differences in weaner mortality were observed. For future studies, it would be interesting to extend the observation period in nurseries and add parameters other than mortality.

In the present study, no differences were observed in the litter productivity or the number of the PRRSV‐positive litters at birth. In a previous study,^23^ it was reported that Ct values >30 most likely represented environmental contamination from facilities, sow secretions or infected littermates. In the present study, the average Ct values of UC were >34, indicating probable contamination from the environment. Only one positive litter at birth per group was related to the presence of positive animals at weaning would strengthen this hypothesis. Another consideration is that, given similar infectious pressures in PG and C groups at birth, the transmission was less efficient in the PG group than in the C group, suggesting that protection after birth was improved in PG. It is worth noting that in both farms the circulating virus was always identified as wild type.

In the present work, sows received the IV only in one gestation. Given the results, it is reasonable to think that repeated cycles of vaccination may have increased the observed differences.

The scheme of vaccination in the present study was a modification of the recommended use of the IV in which the concept of initial vaccination with a MLV was introduced. Both farms maintained the vaccination scheme after the study. At present, Farm 1 remains positive in the nurseries while on Farm 2 PRRSV1 is no longer detected.

It is important to comment on the exclusion of batch #5 in Farm 1 because of a diarrhoea outbreak unrelated to PRRSV. Since the impact of the outbreak was different in the PG and C groups we considered excluding this batch. It is not possible to report the levels of PRRSV antibodies of the dead animals. Therefore, we thought that the most reasonable approach was to exclude it from the analysis and to show the results in Supporting Information.

In the case of Farm 2, in batch #3 an increase in the incidence of PRRSV was noticed in both groups and the percentage of seropositive piglets at weaning dropped. One possible explanation is a problem related to the vaccination although we have no evidence. Sows were vaccinated with the MLV in mid‐July and with PG in mid‐August, with environmental temperatures reaching as high as 34°C–39°C on the days of vaccination. Ventilation was managed by manually opening windows and we cannot discard some effect from thermal stress.

With current knowledge, it is not possible to know if pre‐farrowing vaccination with an MLV would have produced similar results to the ones obtained with the IV. However, the use of a MLV at 90 days of gestation may raise safety concerns (e.g. the vertical transmission of the MLV or a decrease in the number of piglets born alive).^24^

It is important to comment that since the field strains could not be adapted to culture with MARC‐145, despite being easily isolated in macrophages, the neutralisation results were produced using the resident MLV strain. Hence, we cannot be sure how effective the antibodies were for neutralising the specific farm strain. Even so, it can be considered that the higher the NA titres the higher the probability of heterologous neutralisation.

The present study suggests that IV can be used for boosting previous immunity in sows and can be helpful to homogenise the transfer of PRRSV‐specific MDA. A larger study would be needed to assess the precise productive and economic impact under different circumstances.

## CONFLICTS OF INTEREST

Preben Mortensen, Nicolas Guerra, Melanie Sno and Timea Barna work for Ceva Santé Animale. The other authors have not declared any conflicts of interest.

## FUNDING INFORMATION

CEVA Santé Animale.

## ETHICS STATEMENT

All the procedures were approved by the Ethics Committee in Animal and Human Research of the Universitat Autònoma de Barcelona (approval numbers 3221‐CEEA‐UAB and CEEAH‐5691 for the project and the procedure, respectively).

## Supporting information

Supporting Information S1: Number of animals that could be followed up from weaning to the end of nursery phase (9 weeks of age).

Supporting Information S2: Distribution of titres in the viral neutralisation assay.

Supporting Information S3: Summary of the results per farm, batch and group of the main reproductive parameters.

Supporting Information S4: Percentage of deaths for Farms 1 and 2 in the different batches for PG and C groups (1.PG means batch #1 of PG group, 1.C means batch #1 for C group).

Additional‐information‐authors‐and‐funding

## Data Availability

The datasets used and/or analysed during the current study are available from the corresponding author on reasonable request.
