# Supplementary material for: The use of a whole inactivated PRRS virus vaccine administered in sows and impact on maternally derived immunity and timing of PRRS virus infection in piglets
Source: Vet Rec Open. 2022 Apr 5;9(1):e34. doi: 10.1002/vro2.34 (PMC8982505; doi:10.1002/vro2.34)
Supplement: Supplementary file 1 — Supporting Information S1: Number of animals that could be followed up from weaning to the end of nursery phase (9 weeks of age). [file VRO2-9-e34-s001.docx]

|  |  |  | **PG** |  |  |  |  |  |  | **C** |  |  |  |  |  |
| --- | --- | --- | --- | --- | --- | --- | --- | --- | --- | --- | --- | --- | --- | --- | --- |
|  |  | **WoA** | **Pres** | **Susc** | **Inf** | **NI** | **Inc (%)** | **CI (%)** |  | **Pres** | **Susc** | **Inf** | **NI** | **Inc (%)** | **CI (%)** |
| **F1** | **B1** | **4** | 43 | 43 | 6 | 6 | 13,95 | 13,95 |  | 31 | 31 | 5 | 5 | 16,13 | 16,13 |
|  |  | **6** | 43 | 37 | 4 | 1 | 2,70 | 16,28 |  | 31 | 26 | 7 | 5 | 19,23 | 32,26 |
|  |  | **9** | 43 | 36 | 13 | 11 | 30,56 | 41,86 |  | 31 | 21 | 7 | 2 | 9,52 | 38,71 |
|  | **B2** | **4** | 30 | 30 | 1 | 1 | 3,33 | 3,33 |  | 30 | 30 | 1 | 1 | 3,33 | 3,33 |
|  |  | **6** | 30 | 29 | 0 | 0 | 0,00 | 3,33 |  | 30 | 29 | 0 | 0 | 0,00 | 3,33 |
|  |  | **9** | 30 | 29 | 25 | 25 | 86,21 | 86,67 |  | 30 | 29 | 27 | 27 | 93,10 | 93,33 |
|  | **B3** | **4** | 47 | 47 | 2 | 2 | 4,26 | 4,26 |  | 49 | 49 | 12 | 12 | 24,49 | 24,49 |
|  |  | **6** | 47 | 45 | 4 | 4 | 8,89 | 12,77 |  | 49 | 37 | 0 | 0 | 0,00 | 24,49 |
|  |  | **9** | 47 | 45 | 43 | 37 | 82,22 | 91,49 |  | 49 | 37 | 14 | 14 | 37,84 | 53,06 |
|  | **B4** | **4** | 32 | 32 | 1 | 1 | 3,13 | 3,13 |  | 32 | 32 | 2 | 2 | 6,25 | 6,25 |
|  |  | **6** | 32 | 31 | 0 | 0 | 0,00 | 3,13 |  | 32 | 30 | 2 | 2 | 6,67 | 12,50 |
|  |  | **9** | 32 | 31 | 4 | 4 | 12,90 | 15,63 |  | 32 | 28 | 6 | 5 | 17,86 | 28,13 |
|  | **B5** | **4** | 39 | 39 | 6 | 6 | 15,38 | 15,38 |  | 42 | 42 | 3 | 3 | 7,14 | 7,14 |
|  |  | **6** | 39 | 33 | 8 | 5 | 15,15 | 28,21 |  | 42 | 39 | 17 | 17 | 43,59 | 47,62 |
|  |  | **9** | 39 | 25 | 35 | 21 | 84,00 | 82,05 |  | 42 | 22 | 41 | 20 | 90,91 | 95,24 |
|  | **B6** | **3** | 45 | 45 | 1 | 1 | 2,22 | 2,22 |  | 47 | 47 | 2 | 2 | 4,26 | 4,26 |
|  |  | **6** | 44 | 43 | 9 | 8 | 18,60 | 20,00 |  | 46 | 45 | 10 | 10 | 22,22 | 25,53 |
|  |  | **9** | 44 | 35 | 12 | 10 | 28,57 | 42,22 |  | 46 | 35 | 44 | 34 | 97,14 | 97,87 |
|  |  |  |  |  |  |  |  |  |  |  |  |  |  |  |  |
| **F2** | **B1** | **3** | 83 | 83 | 2 | 2 | 2,41 | 2,41 |  | 85 | 85 | 10 | 10 | 11,76 | 11,76 |
|  |  | **6** | 83 | 81 | 13 | 11 | 13,58 | 15,66 |  | 85 | 75 | 30 | 22 | 29,33 | 37,65 |
|  |  | **9** | 82 | 70 | 78 | 65 | 92,86 | 93,98 |  | 83 | 53 | 65 | 48 | 90,57 | 94,12 |
|  | **B2** | **3** | 85 | 85 | 0 | 0 | 0,00 | 0,00 |  | 85 | 85 | 0 | 0 | 0,00 | 0,00 |
|  |  | **6** | 85 | 85 | 6 | 6 | 7,06 | 7,06 |  | 85 | 85 | 6 | 6 | 7,06 | 7,06 |
|  |  | **9** | 85 | 79 | 14 | 10 | 12,66 | 18,82 |  | 85 | 79 | 34 | 29 | 36,71 | 41,18 |
|  | **B3** | **3** | 100 | 100 | 9 | 9 | 9,00 | 9,00 |  | 87 | 87 | 9 | 9 | 10,34 | 10,34 |
|  |  | **6** | 99 | 90 | 90 | 90 | 100,00 | 99,00 |  | 85 | 78 | 83 | 74 | 94,87 | 95,40 |
|  |  | **9** | 87 | 0 | 87 | 0 | 0,00 | 99,00 |  | 75 | 0 | 75 | 2 | 0,00 | 97,70 |

**Additional file 1.** Number of animals that could be followed up from weaning to the end of nursery phase (9 weeks of age). In the table, number of present animals (Present), susceptible (susc), infected (inf), new infections (NI), incidence (Inc) and cumulative incidence (CI) are shown.
