# Supplementary material for: The use of a whole inactivated PRRS virus vaccine administered in sows and impact on maternally derived immunity and timing of PRRS virus infection in piglets
Source: Vet Rec Open. 2022 Apr 5;9(1):e34. doi: 10.1002/vro2.34 (PMC8982505; doi:10.1002/vro2.34)

**Additional file 2.** Distribution of titers in the viral neutralization assay. Results are shown as log2 of the titer.

1.
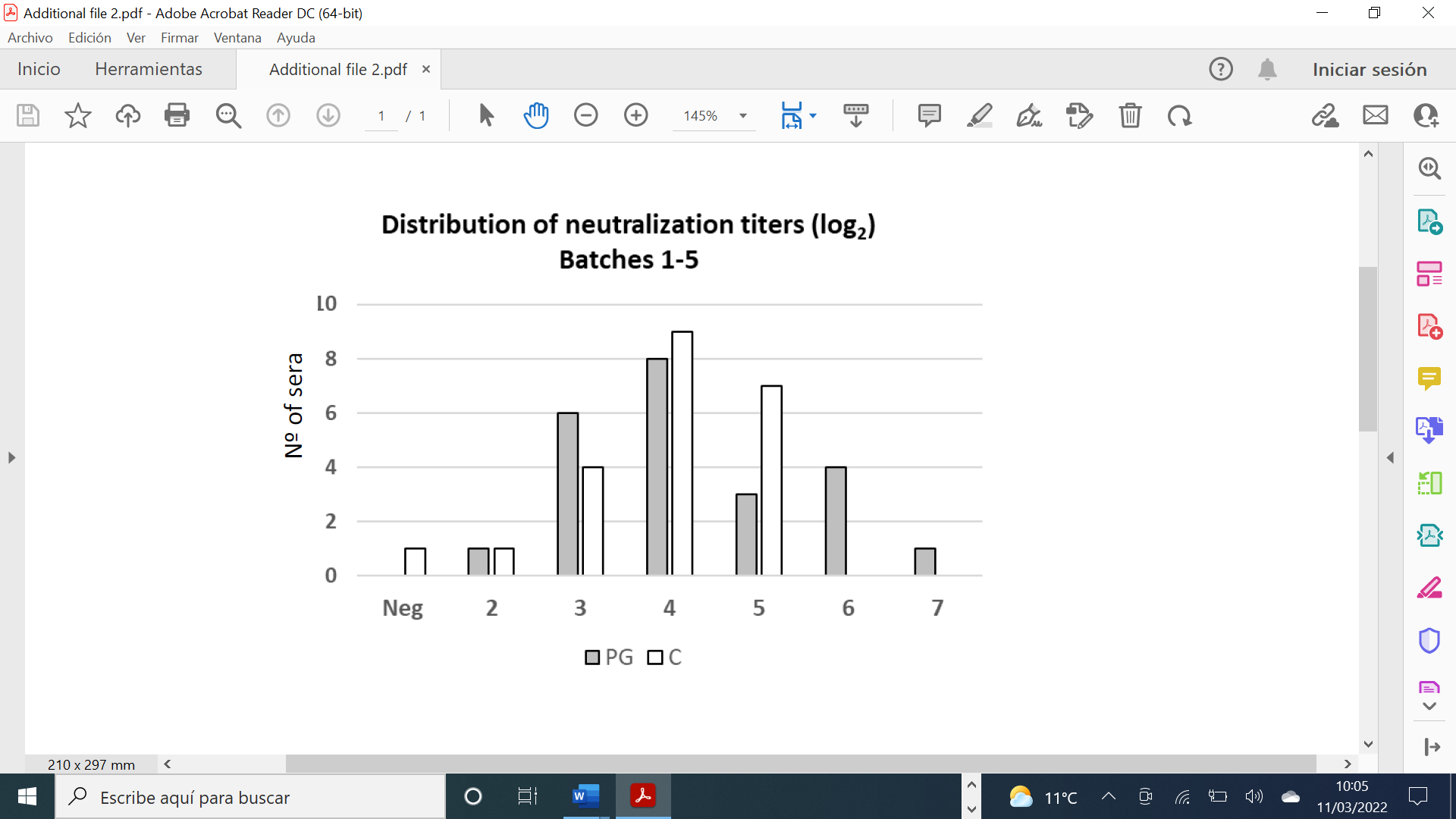
FARM 1
2. FARM 2


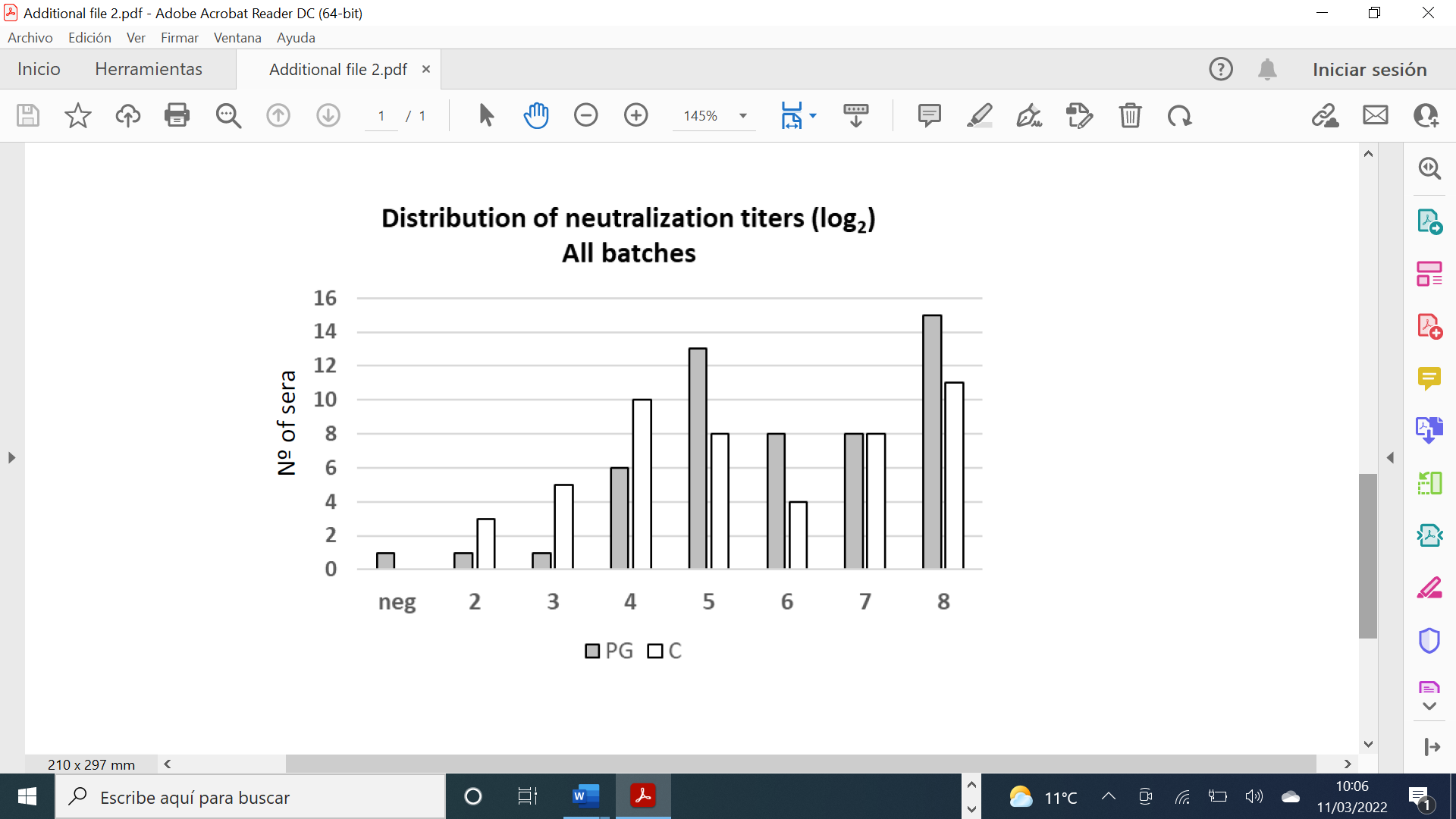

Supplement: Supplementary file 2 — Supporting Information S2: Distribution of titres in the viral neutralisation assay. [file VRO2-9-e34-s005.docx]
