# Supplementary material for: The use of a whole inactivated PRRS virus vaccine administered in sows and impact on maternally derived immunity and timing of PRRS virus infection in piglets
Source: Vet Rec Open. 2022 Apr 5;9(1):e34. doi: 10.1002/vro2.34 (PMC8982505; doi:10.1002/vro2.34)
Supplement: Supplementary file 3 — Supporting Information S3: Summary of the results per farm, batch and group of the main reproductive parameters. [file VRO2-9-e34-s003.docx]

|  | **Batch nº** | **Born alive/litter** | **Mummies/litter** | **Stillborns/litter** | **Weaned/litter** |
| --- | --- | --- | --- | --- | --- |
| **Farm 1** | **1** | PG: 15.1±2.0  C: 14.1±4.1 | PG: 0.5±0.7  C: 0.3±0.7 | PG: 1.1±1.1  C: 1.4±2.2 | PG: 11.9±1.2  C: 11.4±3.2 |
|  | **2** | PG: 13.8±2.3  C: 13.1±2.9 | PG: 0.4±0.6  C: 0.4±0.6 | PG: 0.9±1.1  C: 0.7±0.9 | **PG: 12.3±1.2***  C: 11.5±1.1 |
|  | **3** | PG: 12.6±3.6  C: 12.5±3.5 | PG: 1.4±3.4  C: 0.6±0.9 | PG: 0.7±1.0  C: 1.2±1.8 | PG: 10.8±2.9  C: 9.6±4.1 |
|  | **4** | PG: 13.8±2.8 | PG:1.8±0.9 | PG:1.7±0.8 | PG:12.0±1.2 |
|  |  | C: 13.2±3.0 | C: 2.3±1.9 | C:1.8±0.9 | C:10.93±3.3 |
|  | **5** | PG: 13.2.0±3.5 | PG: 1.5±1.5 | PG: 0.6±1.3 | PG: 10.0±1.07 |
|  |  | C: 13.2±3.7 | C:1.6±1.3 | C: 0.7±0.9 | **C: 11.5±1.5*** |
|  | **6***** | PG: 11.7±2.7 | PG: 1.5±0.7 | PG: 0 | PG: 11±1.15 |
|  |  | C: 14.1±2.0 | C: 1.5±1 | C: 1.6±0.5 | C: 12.4±0.5 |
|  | **Global first 4 batches** | PG: 13.7±2.9  C: 13.2±3.6 | PG: 0.7±2.2  C: 0.4±0.8 | PG: 0.9±1.1  C: 1.1±1.6 | **PG: 11.7±2.1****  C: 10.8±3.2 |
|  | **Global all** | PG:13.61±2.6  C:13.3±3.4 | PG: 0.49±0.7  C:0.53±0.9 | PG:1.07±1.2  C:1.23±0.9 | PG: 11.57±1.4  C: 11.1±2.7 |
| **Farm 2** | **1** | PG: 13.3±3.1  C: 12.2±2.3 | PG: 0.04±0.2  C: 0.08±0.3 | PG: 0.4±0.7  C: 0.5±0.7 | PG: 11.5±1.7  C: 10.8±1.2 |
|  | **2** | PG: 12.7±3.1  C: 12.2±3.6 | PG: 0.0±0.0  C: 0.0±0.0 | PG: 0.5±0.8  C: 0.8±1.5 | PG: 11.1±1.3  C: 10.9±1.4 |
|  | **3** | PG: 13.5±3.2  C: 13.5±2.6 | PG: 0.1±0.4  C: 0.04±0.2 | PG: 0.9±1.2  C: 0.9±1.0 | PG: 10.8±2.3  C: 11.2±2.8 |
|  | **Global all** | PG:13.2±3.1  C:12.6±3.1 | PG: 0.05±0.2  C:0.04±0.2 | PG:0.64±0.9  C:0.7±1.2 | PG: 11.13±1.9  C: 10.99±1.9 |

**Additional file 3.** Summary of the results per farm, batch and group of the main reproductive paramenters

*p<0.05

**p=0.05

***It has to be considered that only 7 sows were evaluated per group (6 primiparous and 1 multiparous per group)
