# Supplementary material for: The use of a whole inactivated PRRS virus vaccine administered in sows and impact on maternally derived immunity and timing of PRRS virus infection in piglets
Source: Vet Rec Open. 2022 Apr 5;9(1):e34. doi: 10.1002/vro2.34 (PMC8982505; doi:10.1002/vro2.34)
Supplement: Supplementary file 4 — Supporting Information S4: Percentage of deaths for Farms 1 and 2 in the different batches for PG and C groups (1.PG means batch #1 of PG group, 1.C means batch #1 for C group). [file VRO2-9-e34-s002.docx]

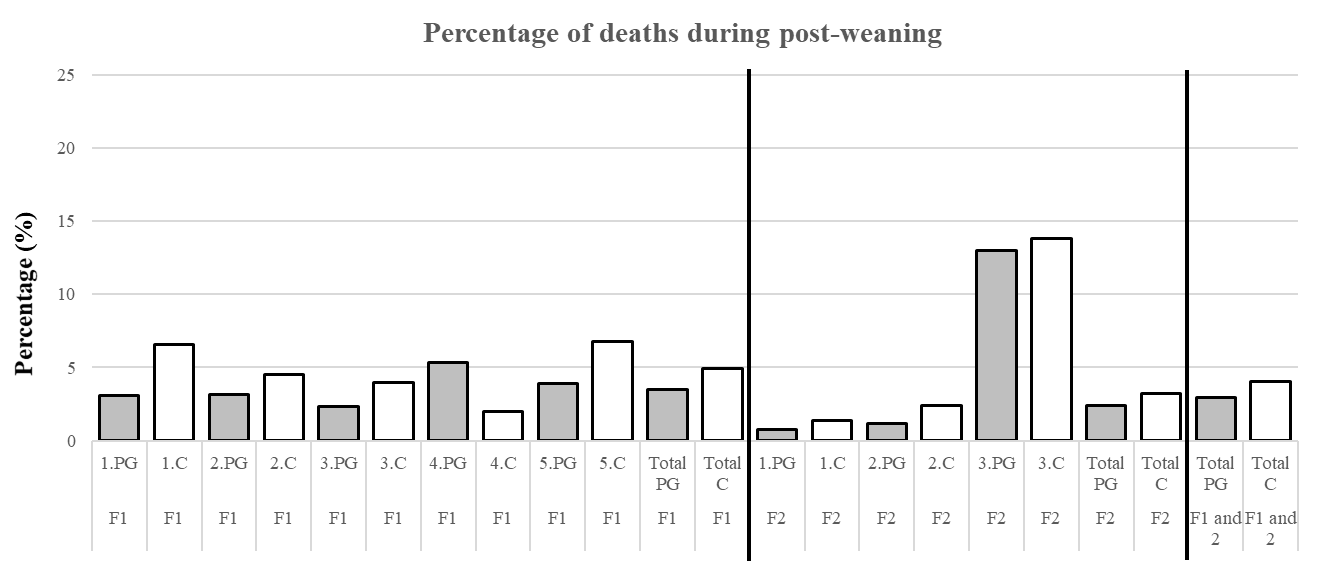
**Additional file 4.** Percentage of deaths for farm 1 (F1) and farm 2 (F2) in the different batches for PG and C groups (1.PG means batch 1 of PG group, 1.C means batch 1 for C group). No deaths were registered for batch number 6 in F1.
