# Supplementary material for: The use of a whole inactivated PRRS virus vaccine administered in sows and impact on maternally derived immunity and timing of PRRS virus infection in piglets
Source: Vet Rec Open. 2022 Apr 5;9(1):e34. doi: 10.1002/vro2.34 (PMC8982505; doi:10.1002/vro2.34)
Supplement: Supplementary file 5 — Additional‐information‐authors‐and‐funding [file VRO2-9-e34-s004.docx]

**Authors contribution statement**

Design of the trial was done by EM, GEM-V, PM and NG. Samples and productive data were collected and recorded by GEM-V, HC, MC and MT. Serological and virological analysis were performed by GEM-V, HC, YL, MC and EM. Statistical analysis was performed by EM and GEM-V. All authors contributed to the manuscript.

**Funding information**

The present paper was funded by CEVA santé animale.
